# A Novel Guanine Elicitor Stimulates Immunity in *Arabidopsis* and Rice by Ethylene and Jasmonic Acid Signaling Pathways

**DOI:** 10.3389/fpls.2022.841228

**Published:** 2022-02-17

**Authors:** Lulu Wang, Haoqi Liu, Ziyi Yin, Yang Li, Chongchong Lu, Qingbin Wang, Xinhua Ding

**Affiliations:** ^1^State Key Laboratory of Crop Biology, Shandong Provincial Key Laboratory of Agricultural Microbiology, College of Plant Protection, Shandong Agricultural University, Tai’an, China; ^2^Shandong Pengbo Biotechnology Co., Ltd., Tai’an, China

**Keywords:** elicitor, ethylene (ET) signaling, guanine, jasmonic acid (JA) signaling, nucleobases, rice sheath blight (ShB)

## Abstract

Rice sheath blight (ShB) caused by *Rhizoctonia solani* is one of the most destructive diseases in rice. Fungicides are widely used to control ShB in agriculture. However, decades of excessive traditional fungicide use have led to environmental pollution and increased pathogen resistance. Generally, plant elicitors are regarded as environmentally friendly biological pesticides that enhance plant disease resistance by triggering plant immunity. Previously, we identified that the plant immune inducer ZhiNengCong (ZNC), a crude extract of the endophyte, has high activity and a strong ability to protect plants against pathogens. Here, we further found that guanine, which had a significant effect on inducing plant resistance to pathogens, might be an active component of ZNC. In our study, guanine activated bursts of reactive oxygen species, callose deposition and mitogen-activated protein kinase phosphorylation. Moreover, guanine-induced plant resistance to pathogens depends on ethylene and jasmonic acid but is independent of the salicylic acid signaling pathway. Most importantly, guanine functions as a new plant elicitor with broad-spectrum resistance to activate plant immunity, providing an efficient and environmentally friendly biological elicitor for bacterial and fungal disease biocontrol.

## Introduction

Rice is one of the most important food crops in the world, and the future demands for rice will be higher due to the trend of increasing populations worldwide. Plant pathogens not only reduce rice yield but also produce toxins, resulting in food contamination, which further threatens human health. Rice sheath blight (ShB) is caused by the necrotrophic fungus *Rhizoctonia solani* (*R. solani*); ShB is one of the most destructive diseases and a serious threat to the stability of rice production worldwide (Sathe et al., 2021). The cultivation of high-yielding varieties and heavy use of nitrogen fertilizers have caused a sharp rise in ShB prevalence (Savary et al., 1995). ShB can develop water-soaked, oval-shaped or irregularly elongated spots on leaf sheaths and leaf blades that are infected by hyphae or sclerotia (Lin et al., 2020; Molla et al., 2020). In China, the straw return policy returns almost all sclerotia produced back to rice fields. In addition, no effective ShB-resistant cultivars have been applied to rice agricultural production to date (Chen et al., 2012; Molla et al., 2020). At present, chemical fungicides are widely used to minimize the damage caused by ShB; however, pathogens that are resistant to fungicides have emerged (Casida and Durkin, 2017; Singh et al., 2019; Abbas et al., 2021), and these pathogens markedly reduce chemical fungicide efficacy. To make matters worse, chemical pesticides pollute the soil, water resources and crops, posing risks to human health and the ecological environment (Rodriguez-Salus et al., 2016; Casida and Durkin, 2017; Kim et al., 2017). Together, these challenges require pollution-free and efficient biological fungicides to control ShB.

In previous study, fungal biocontrol agents derived from *Aspergillus*, *Clonostachys*, *Chaetomium*, *Trichoderma*, and *Streptoverticillium* were used for seed germination pretreatment, root dipping and foliar application (Park et al., 2002, 2005; Naeimi et al., 2010; Shasmita et al., 2019). Unfortunately, no effective commercial biocontrol agent has been successfully used for ShB control thus far. To reduce the use of chemical fungicides, plant immune elicitors (PIEs) have become developing trend in elicitor-based biopesticide biopesticides (Schwessinger and Ronald, 2012). The most important feature of PIEs is their activation of plant immunity instead of killing pathogens directly, in contrast to traditional chemical fungicides. Generally, PIEs are classified as oligosaccharides, peptides, proteins, lipids and small-molecule metabolite elicitors (Schwessinger and Ronald, 2012; Yakhin et al., 2016). For instance, the lipid elicitor lipopolysaccharide, protein-like elicitor BcGs1 and carbohydrate elicitor β-glucan can protect plants against pathogen invasion by triggering innate plant immunity (Yamaguchi et al., 2000; Ma et al., 2015; Ranf et al., 2015).

Many studies have shown that PIEs enhance plant immunity by directly or indirectly activating the signal transduction pathway of plant endogenous hormones such as jasmonic acid (JA), salicylic acid (SA), and ethylene (ET). For instance, copper ions (Cu^2+^) can trigger plant immunity by upregulating *AtACS8* gene expression through CuRE *cis*-acting elements, further promoting the early biosynthesis of ethylene and promoting the development of the ethylene pathway in *Arabidopsis* (Liu et al., 2015; Zhang et al., 2018). In addition, rutin enhances plant resistance to bacterial pathogens by activating the SA signaling pathway (Yang et al., 2016). A recent study has shown that silicon can enhance the resistance of potatoes to late blight, which depends on the JA/ET signaling pathway (Xue et al., 2021). Validamycin A induced broad-spectrum disease resistance in both dicots and monocots through the SA and JA/ET signaling pathways (Bian et al., 2020). ET and JA are widely involved in the interactions between plants and pathogens. JA activates plant resistance to necrotrophic pathogens and chewing insects (Howe and Jander, 2008; Brenya et al., 2020). In tomatoes, *MYC2* positively regulates pathogen-responsive genes, thereby improving plant resistance to *Botrytis cinerea* (Du et al., 2017). The *ERF1* and *ERF6* ethylene-responsive factors can enhance the resistance of *Arabidopsis* to the soilborne fungus *Fusarium oxysporum* and the necrotrophic fungus powdery mildew, respectively (Berrocal-Lobo and Molina, 2004; Meng et al., 2013). *TaCRK3* mediates wheat resistance to *Rhizoctonia cerealis* by enhancing the expression of genes involved in ET signaling pathway defense (Guo et al., 2021).

Nucleotide metabolism is a fundamental biological process in many organisms and plays a vital role in plant development and plant responses to biotic and abiotic stresses (Dahncke and Witte, 2013). A recent study has shown that nucleotides are also involved in plant immune responses. Both cyclic GMP (cGMP) and cyclic nucleotide monophosphates (cNMPs) are essential secondary messengers in plants and have been reported to be involved in plant immune responses (Gehring and Turek, 2017; Isner and Maathuis, 2018). In addition, nicotinamide adenine dinucleotide (NAD) is a common electronic carrier involved in the plant response to pathogen invasion, extracellular NAD-induced transcriptional changes and disease resistance to citrus canker (Noctor et al., 2006; Hashida et al., 2009; Alferez et al., 2018). Similarly, adenosine-5′-triphosphate (ATP) is the most direct energy source in most organisms, and extracellular ATP (eATP) can be recognized by the receptor-like kinase DORN1 located on the surface of the plant cell membrane, inducing immune responses such as calcium ion (Ca^2+^) bursts and mitogen-activated protein kinase (MAPK) phosphorylation in plants. Furthermore, eATP can improve plant resistance to *Botrytis cinerea* by activating JA signaling (Tripathi et al., 2018). A previous study has shown that guanosine phosphate nucleotides play a significant role in affecting plant–pathogen interactions and responses to plant hormones, such as SA, ET, jasmonic acid JA, and abscisic acid (ABA; Takahashi et al., 2004; Abdelkefi et al., 2018). Nevertheless, the role of nucleobases in regulating plant immunity has not been reported.

Guanine is one of the primary components of DNA. Previous research on guanine has been conducted mainly in the medical field. Guanine is the most easily oxidized base. Guanine oxidation produces 8-oxoguanine, which can pair with adenine and induce guanine-thymine mutations in DNA (Freudenthal et al., 2015). A guanine analog, favipiravir, was approved for influenza treatment and can effectively inhibit the RNA-dependent RNA polymerase of RNA viruses such as influenza, Ebola and COVID-19 (Furuta et al., 2017; Ghasemnejad-Berenji and Pashapour, 2021). In this study, we found that guanine enhanced plant resistance to pathogens and induced reactive oxygen species bursts, callose deposition and MAPK phosphorylation. Comprehensive transcriptome analysis and the transcription of signaling pathway genes with guanine-induced plant disease resistance were analyzed, and further examination found that guanine failed to activate rice resistance to ShB in the *COI1b-RNAi* plants and *etr2*, *etr3* mutant plants. Moreover, we propose that guanine protected rice against ShB invasion depending on the ET receptor genes *OsETR2* and *OsETR3* and JA receptor gene *OsCOI1b*. In addition, guanine enhanced *Arabidopsis* resistance to *Pst* DC3000 and depended on *AtETR1*, *AtEIN2*, and *AtJAR1*. These results suggest that guanine induced plant immunity depending on JA and ET signaling. Our study preliminarily revealed the molecular mechanism by which guanine not only activates plant immunity but also provides an environmentally friendly, efficient prevention and control strategy for many diseases, including ShB.

## Materials and Methods

### Plant Materials and Guanine Treatments

In this study, the japonica rice (*Oryza sativa*) cultivar (Zhonghua 11, ZH11) was used as the wild type in rice experiments, the T-DNA insertion mutants *etr2* and *etr3* had a Dongjin (DJ) background, and the RNA interference lines *COI1b-RNAi* and SA-deficient *NahG* transgenic plants had a Nipponbare (NIP) background. *Arabidopsis thaliana* ecotype Columbia (Col-0) was used as a wild-type control in *Arabidopsis* experiments, and all *Arabidopsis* mutants used in this research had a Col-0 background. All rice plants were grown at 28°C with 75% relative humidity and a 16/8 h light/dark photoperiod. All *Arabidopsis* seedlings were grown at 22°C with 70% relative humidity and a 12/12 h light/dark photoperiod. In our study, 4-week-old rice or *Arabidopsis* plants were sprayed with guanine solution containing 0.05% silwet-L 77 or 0.05% Tween-20, respectively.

### Pathogen Infection Experiments

The *R. solani* strain YWK196 was incubated on potato dextrose agar (PDA) medium at 28°C for 2 days, transferred to potato dextrose medium containing matchsticks and grown at 28°C until the mycelium was wrapped around the matchstick. The matchsticks with hyphae were inserted into plant sheaths after guanine treatment for 2 h, and the lesion length was recorded at 3–5 days postinoculation in the field. Leaf inoculation with YWK196 involved the following steps. The clumps of *R. solani* were placed onto the middle of leaves, which were kept moist in a square petri dish (30 cm × 30 cm). The lesion areas were photographed and measured by ImageJ.^1^ Inoculation of *Xanthomonas oryzae* pv. *oryzicola* (Xoc) strain RS105, which causes bacterial leaf streak in rice, and *Xanthomonas oryzae* pv. *oryzae* (Xoo) strain PX099A (the causative agent of bacterial blight in rice) was performed according to previously reported methods (Ju et al., 2017; Li et al., 2018). *Pseudomonas syringae* pv. tomato (*Pst*) strain DC3000 was cultured on potato saccharose agar (PSA) medium at 28°C for 24 h. Before inoculation, the bacterial suspension was adjusted to an OD_600_ of 0.002. The bacterial suspension was infiltrated into plant leaves with a sterile needleless syringe after guanine treatment for 2 h. The lesion area was observed, and the bacterial growth assay was performed after 3 days.

### Bacteria Growth Assay

For the quantification of bacteria, six diseased leaves from different plants were harvested and weighed. Then, the diseased leaves were surface sterilized with 75% alcohol for 1 min and washed with sterile distilled water three times. After that, the diseased leaves were ground with a mortar, and a gradient dilution (1:10) of the homogenate was performed. Finally, 10 μL of homogenate was plated on PSA medium and cultured at 28°C, and the number of colonies was calculated after 24 h.

### Callose Deposition

Following guanine treatment for 24 h, 4-week-old rice or *Arabidopsis* leaves were harvested and put into ethanol:phenol [3:1 (v/v)] solution, vacuumed for 30 min, and then transferred into a water bath at 60°C until the leaves became white and transparent. After being washed with water three times, the samples were stained in aniline blue solution (0.1% w/v aniline blue in 150 mM K_2_HPO_4_, pH = 9.5) in the dark overnight at room temperature, and then the samples were washed with deionized water three times to remove excess dye solution. Callose deposition was photographed under a fluorescence microscope.

### Mitogen-Activated Protein Kinase Detection

Four-week-old *Arabidopsis* seedlings were selected for protein extraction after guanine treatment for 0, 10, 30, and 60 min. First, leaf samples were rapidly frozen in liquid nitrogen and ground to fine powder. Protein extraction was performed using plant protein extraction reagent (CWBIO, China) according to the manufacturer’s instructions. In addition, phosphorylated MAP kinases were detected with anti-phospho-p44/42 MAP kinase primary antibodies (Cell Signaling Technology, United States).

### Detection of Hydrogen Peroxide Production

The production of hydrogen peroxide (H_2_O_2_) in leaves was detected using 3,3-diaminobenzidine (DAB) as an indicator. Four-week-old rice and *Arabidopsis* leaves were harvested after 2 h of guanine treatment, and the samples were immediately transferred into a DAB (0.5 mg/mL) staining solution and then vacuum-infiltrated for 30 min. The samples were washed three times with deionized water and then incubated for 8 h under light at room temperature. Subsequently, the samples were decolorized with boiled ethanol. Finally, the samples were imaged by using a stereomicroscope (Lu et al., 2019). To detect ROS production by using H_2_DCFDA under confocal microscopy, the plants were treated with guanine (100 ng/mL) for 0, 2, 4, 8, 12 and 24 h, 10 μM H_2_DCFDA solution was infiltrated into plant leaves, and the fluorescence signal was detected 20 min later using an EnSpire Plate Reader (PerkinElmer, United States) with 502 nm excitation and 530 nm emission.

### RNA Extraction, Reverse Transcription, and Quantitative Real-Time PCR

Rice leaf total RNA was extracted using TRIzol reagent (T9424, Sigma-Aldrich, United States) following the kit instructions provided by the manufacturer. Reverse transcription was performed using HiScript III-RT SuperMix for qPCR (+ gDNA wiper) (Vazyme, China). Genomic DNA was first removed from 1 μg of total RNA using 4 × gDNA wiper mix and then reverse transcribed with 5 × HiScriptIII^®^ qRT SuperMix at 37°C for 15 min and 85°C for 5 s. Quantitative real-time PCR was carried out on the QuantStudio 6 Flex Real-Time PCR System (Life Technologies, United States) according to the method provided in the UltraSYBR Mixture (CWBIO, China) instructions. Expression of the rice endogenous actin gene Actin (Accession No: AY212324) was used for internal analysis of the results. The quantitative qRT-PCR primers are shown in Supplementary Table 1.

### RNA-Seq and Analysis

Four-week-old rice leaves were harvested at 0 h, 2 h, or 24 h after guanine treatment with three replicates of each treatment for a total of nine samples. RNA samples were constructed as previously reported (Feng et al., 2016; Wu et al., 2019), and sequencing was performed with a BGISEQ-500 platform by Wuhan Genomic Institution (^2^ BGI, Shenzhen, China). The expression levels of individual genes were normalized to FKPM (fragments per kilobase million) reads by RNA-seq through the expectation maximization algorithm. All differential gene expression was based on the following standard: the t significantly differential expression of genes was defined with the absolute value of log_2_ ratio≥1 and *P* ≤ 0.05. Heatmap analysis of data was performed *via* the BAR Heatmapper tool^3^.

## Results

### Guanine Enhances Rice Resistance Against Rice Sheath Blight

**FIGURE 1:**
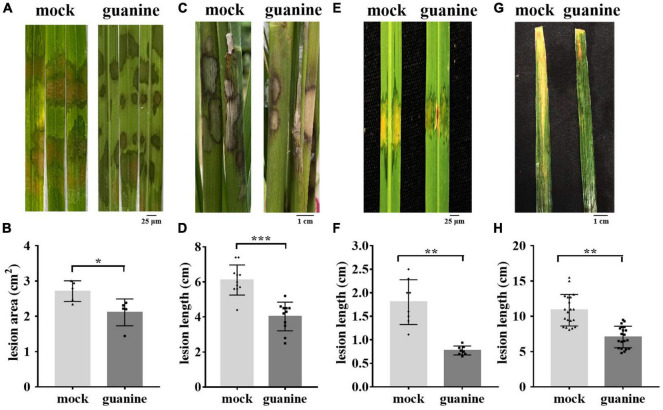
Guanine improved the resistance of rice to *R. solani.* **(A)** Rice sheath blight symptoms on the leaves after indoor guanine treatment. The images were photographed at 3 dpi. **(B)** Lesion area after 3 days of inoculation with *R. solani* after guanine treatment. Error bars indicate the SD (*n* = 5). Asterisks indicate *P* < 0.05 (*) in Student’s *t*-test analysis. **(C)** Phenotypes of the rice sheaths inoculated with *R. solani* after guanine treatment in the field. The images were photographed at 3 dpi (*n* = 11). **(D)** Rice sheath blight disease lesion length 3 days after *R. solani* inoculation after rice sheath guanine treatment. Error bars indicate the SD (*n* = 11). Asterisks indicate *P* < 0.001 (***) in Student’s *t*-test analysis. **(E)** Bacterial blight disease symptoms on rice leaves after guanine treatment. The images were photographed at 10 days postinoculation. **(F)** Treatment with guanine 2 h before inoculation with PXO99A and lesion length measurement at 10 days postinoculation (*n* = 8). Asterisks indicate *P* < 0.01 (**) in Student’s *t*-test analysis. **(G)** Rice leaves treated with 100 ng/mL guanine 2 h before Xoc strain RS105 inoculation. The images were photographed at 15 days postinoculation. **(H)** Lesion lengths after guanine treatment compared with that of the mock at 15 dpi with Xoc strain RS105 (*n* = 20). Asterisks indicate *P* < 0.05 (*), *P* < 0.01 (**), *P* < 0.001 (***), and *P* < 0.001 (***) in Student’s *t*-test analysis. The experiment was repeated three times with similar results.

A previous study showed that ZhiNengCong (ZNC), an extract of the endophyte *Paecilomyces variotii*, can protect plants against pathogenic infection (Lu et al., 2019; Cao et al., 2021). To investigate the effective immune fraction of ZNC, high-performance liquid chromatography (HPLC) and liquid chromatography-mass spectrometry (LC–MS) were performed. Many nucleotides and their derivatives were identified as the active fractions of ZNC, including guanine (Supplementary Table 2). To determine whether guanine stimulates disease resistance in plants and at what concentration, 1 ng/mL, 10 ng/mL, and 100 ng/mL guanine were selected to perform the following experiments. The test was first performed on *Arabidopsis*. Our results showed that guanine treatment enhanced the resistance of *Arabidopsis* to *Pst* DC3000, and the increase in *Pst* DC3000 resistance was consistent with an increase in guanine treatment concentration (Supplementary Figures 1A,B). The lowest effective concentration, 100 ng/mL, was selected for further experiments. To investigate whether guanine can enhance rice resistance to ShB, inoculation experiments with the *R. solani* strain YWK196 were conducted in rice. The results indicated that rice leaves treated with guanine exhibited weaker disease symptoms, which showed shorter lesions and smaller areas of disease in the greenhouse (Figures 1A,B), suggesting that guanine significantly enhanced the resistance of rice to ShB. In the field, the average ShB lesion length on guanine-treated rice showed a 37% reduction compared with that of the mock rice (Figures 1C,D). In addition, 100 ng/mL guanine enhanced the resistance of rice to *Xanthomonas oryzae* pv. *oryzicola* (Xoc) and *Xanthomonas oryzae* pv. *oryzae* (Xoo), which caused rice bacterial leaf streak and bacterial blight, respectively (Figures 1E–H). To further determine whether guanine directly inhibits pathogen growth, pathogen growth experiments were conducted in solid medium containing 0, 1, 10, 100, or 200 ng/mL guanine. As shown in Supplementary Figure 2, guanine failed to inhibit the growth of RS105, PXO99A, YWK196, and *Pst* DC3000, which indicated that guanine conferred plant resistance to pathogens independently of antimicrobial activity. According to these results, we speculated that guanine may enhance disease resistance by stimulating immunity in plants.

### Guanine Activates Plant Immune Responses

**FIGURE 2:**
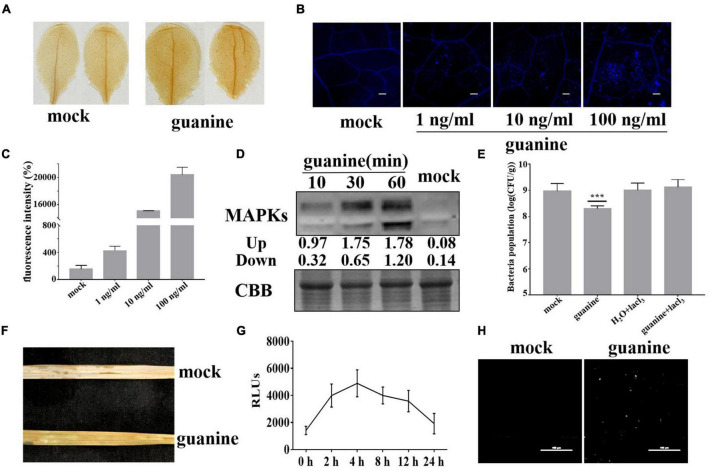
Guanine-induced plant immune responses. **(A)** DAB staining to detect H_2_O_2_ accumulation at 2 hpt after guanine treatment in *Arabidopsis*; brown deposits indicate H_2_O_2_ concentration. **(B)** Guanine-induced callose deposits in *Arabidopsis*. The leaves of 4-week-old Col-0 seedlings were treated with the mock and 1, 10, and 100 ng/mL guanine, and callose deposits were revealed by aniline blue staining. Scale bars = 25 μm. **(C)** Quantification of callose fluorescence intensity of guanine-treated *Arabidopsis* leaves was performed using ImageJ software. Error bars indicate the SD (*n* = 4). **(D)** Guanine-activated MAPK phosphorylation in *Arabidopsis* leaves. MAPK phosphorylation was detected by the phospho-p44/42 MPK antibody at the indicated times. Coomassie brilliant blue (CBB) staining of ribulose-1,5-bis-phosphate carboxylase/oxygenase was used to ensure equal protein loading in each lane. **(E)** Growth population of *Pst* DC3000 in the leaves of *Arabidopsis* plants treated with mock, guanine, H_2_O + lacl_3_ or guanine + lacl_3_. Error bars indicate the SD. Asterisks indicate *P* < 0.001 (***) in Student’s *t*-test analysis. **(F)** Accumulation of H_2_O_2_ in rice leaves after guanine treatment by DAB staining. **(G)** H_2_DCFDA was used to detect ROS accumulation in the rice leaves after guanine treatment at 0, 2, 4, 8, 12 and 24 h. Error bars indicate the SD (*n* = 5). **(H)** Callose deposits in guanine-treated rice leaves after aniline blue staining. The leaves of 4-week-old ZH-11 seedlings were treated with mock and 100 ng/mL guanine and then stained using aniline blue. Scale bars = 100 μm.

Plant elicitors, such as flagellin 22 and Cu^2+^, generally trigger ROS bursts, callose deposition and MAPK phosphorylation in plant leaves (Liu et al., 2015). Thus, we determined whether guanine could induce plant immune responses. H_2_O_2_ is the main component of ROS, and its accumulation can be detected by diaminobenzidine (DAB) staining. In *Arabidopsis*, after guanine treatment, a deeper brown color was observed 2 h posttreatment (hpt) compared to that seen with the mock treatment (Figure 2A). Aniline blue staining was used to determine callose accumulation. As shown in Figures 2B,C, *Arabidopsis* leaves had significantly more callose deposits in response to treatment with guanine compared to the mock treatment (Figures 2B,C). In addition, elicitor-triggered plant immunity is often associated with MAPK phosphorylation activation (Asai et al., 2002), and guanine treatment significantly promoted the phosphorylation of MAPKs compared with the mock treatment at the indicated times (Figure 2D). Ca^2+^ is identified as an essential secondary messenger regulating multitudinous cellular processes (Bleecker and Kende, 2000), and Ca^2+^ influx is the first event in the activation of the defense response in plants. To determine whether Ca^2+^ influx is required for the guanine-induced defense response, a calcium channel blocker, lanthanum chloride (LaCl_3_), was used to treat *Arabidopsis* leaves in combination with guanine. The results showed that using LaCl_3_ significantly reduced guanine-induced disease resistance, implying that Ca^2+^ influx is necessary for the guanine-mediated defense response in *Arabidopsis* (Figure 2E). In rice, guanine also promoted the accumulation of ROS, as shown by DAB staining (Figure 2F). In addition, H_2_DCFDA fluorescence was used to detect ROS production. The results showed that guanine increased the accumulation of ROS within 24 h and peaked at 4 hpt (Figure 2G). The rice leaves showed a significant increase in callose deposition after guanine treatment (Figure 2H).

### Transcriptome Profiling Reveals That Guanine Regulates the Expression of Defense-Associated Genes

**FIGURE 3:**
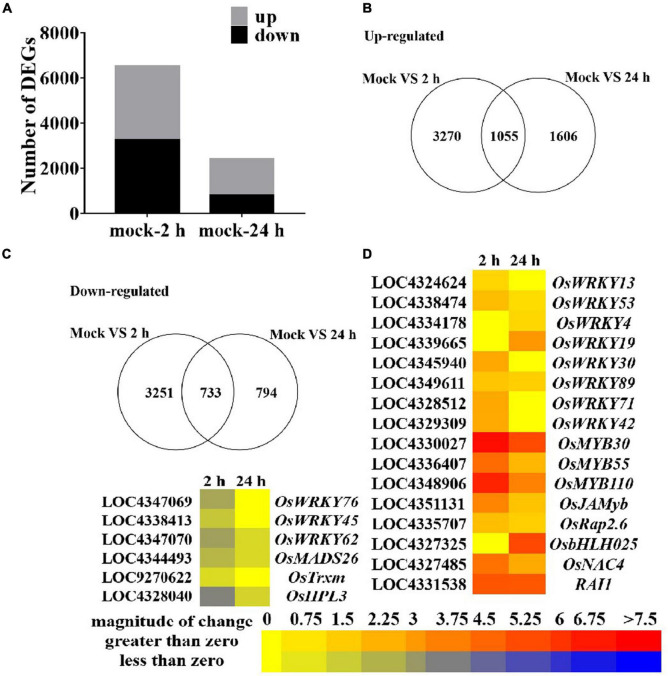
An overview of guanine-regulated gene expression in rice leaves. **(A)** Number of up- and downregulated genes after guanine treatment at 2 and 24 hpt. **(B)** Venn diagram of upregulated genes in the mock vs. guanine-2 h and mock vs. guanine-24 h groups. **(C)** Downregulated expression of genes after 2 and 24 hpt of guanine treatment. **(D)** Heatmap analysis of disease resistance-related gene expression after guanine treatment at 2 and 24 hpt.

To further explore the role of guanine in regulating the expression of genes at the transcriptional level in rice, we performed an RNA sequencing transcriptome analysis of rice leaves treated with guanine (100 ng/mL) at 0, 2, and 24 hpt. The samples were divided into three groups with three biological replicates. Each sample produced at least 44 million high-quality clean reads with a clean date rate of 97% or higher for all samples, and an average of 83.36% reads were mapped to the rice genome, representing a total of 25045 genes expressed in all samples (Supplementary Tables 3, 4). Here, 3270 and 1606 genes were upregulated and 3251 and 794 genes were downregulated at 2 and 24 h after guanine treatment, respectively (Figures 3A–C). A set of differentially expressed genes (DEGs) that negatively regulate defense responses [namely, *OsWRKY76* (Yokotani et al., 2013), *OsWRKY45* (Tao et al., 2009), *OsWRKY62* (Liang et al., 2017), *OsMADS26* (Khong et al., 2015), *OsTrxm* (Hu et al., 2021), and *OsHPL3* (Hu et al., 2021)] were found to be significantly downregulated with guanine treatment. Genes [namely, *OsWRKY13* (Xiao et al., 2013), *OsWRKY53* (Chujo et al., 2007), *OsWRKY4* (Wang et al., 2015), *OsWRKY19* (Du et al., 2021), *OsWRKY30* (Peng et al., 2012), *OsWRKY89* (Wang et al., 2007), *OsWRKY71* (Liu et al., 2007), *OsWRKY42* (Cheng et al., 2015), *OsMYB30* (Li et al., 2020), *OsMYB55* (Kishi-Kaboshi et al., 2018), *OsMYB110* (Kishi-Kaboshi et al., 2018), *OsJAMyb* (Cao et al., 2015), *OsRap2.6* (Wamaitha et al., 2012), *OsNAC4* (Kaneda et al., 2009), and *RAI1* (Kim et al., 2012)] that are involved in the positive regulation of plant disease resistance responses were markedly upregulated after guanine treatment (Figure 3D). These results showed that guanine could regulate the expression of disease resistance-related genes in rice.

### Guanine-Induced Plant Disease Resistance Is Dependent on the Ethylene and Jasmonic Acid Signaling Pathways

**FIGURE 4:**
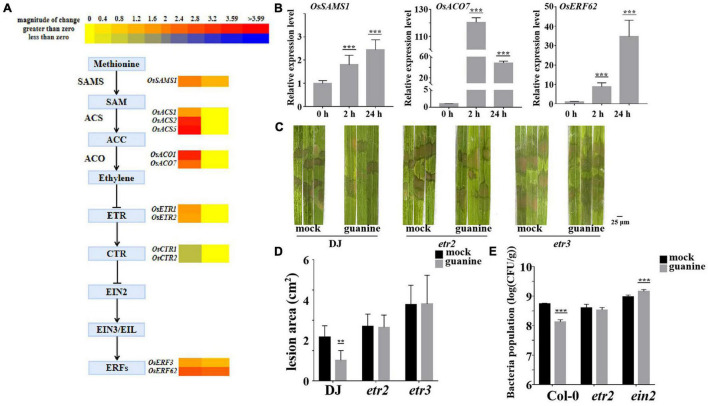
Guanine activated ET signaling pathway gene expression and enhanced plant disease resistance dependent on ethylene receptors. **(A)** Heatmap analysis of ET biosynthesis and response gene expression after guanine treatment at 2 and 24 hpt. **(B)** qRT-PCR for the detection of ethylene pathway genes *OsSAMS1*, *OsACO7*, and *OsERF62.* The horizontal coordinates indicate the time of guanine- and mock-treated rice, and the vertical coordinates indicate the relative gene expression levels of guanine compared to the mock at the same time point. **(C)** Susceptible phenotype of the *etr2* and *etr3* mutant plants. Leaves of the rice plants treated with the mock and 100 ng/mL guanine prior to *R. solani* strain YWK196 inoculation. The disease symptoms were photographed at 3 dpi. **(D)** Measurement of the area of **(C)** by ImageJ. Asterisks indicate *P* < 0.01 (**) in Student’s *t*-test analysis. **(E)** Bacterial population in Col-0, *etr1* and *ein2* plant leaves. *Arabidopsis* leaves treated with the mock and 100 ng/mL guanine prior to *Pst* DC3000 inoculation. The bacterial population was calculated at 3 dpi. Asterisks indicate *P* < 0.001 (***) in Student’s *t*-test analysis.

To further investigate the molecular mechanism of guanine-induced disease resistance in rice, multiple disease signaling pathways were analyzed. The results showed that gene expression in the ET, JA, and SA signaling pathways was significantly upregulated. ET-JA and SA signaling pathways play an essential role in plant defense against biotrophic and necrotrophic pathogens (McDowell and Dangl, 2000; Berens et al., 2017; Zhao Z. X. et al., 2021). RNA sequencing analysis suggested that ethylene biosynthesis gene expression was upregulated by guanine at 2 and 24 hpt (Figure 4A), including *OsSASM1*, *OsACS1*, *OsACS2*, *OsACS5*, *OsACO1*, and *OsACO7* (Li et al., 2011). Similarly, guanine increased the expression of ethylene-responsive transcriptional genes such as *OsERF3* and *OsERF62* (Hu et al., 2008; Lu et al., 2011; Figure 4A). Furthermore, as shown in Figure 4B, qRT-PCR experiments demonstrated that guanine treatment upregulated the expression of *OsSASM1*, *OsACO7*, and *OsERF62*, which was consistent with the RNA-seq data (Figure 4B). In rice, *OsERS1*, *OsERS2*, *OsETR2*, *OsETR3*, and *OsETR4* have been identified as ET receptors (Wuriyanghan et al., 2009; Zhao H. et al., 2021). Here, the ET receptor T-DNA insertion mutants *etr2* and *etr3* were used to determine whether guanine induced rice resistance to *R. solani* that relies on ET signaling. As shown in Figures 4C,D, the lesion area was significantly smaller after guanine treatment compared to that in the mock in wild-type plants Dongjin (DJ); however, a similar lesion area between the mock and guanine treatment groups in *etr2* and *etr3* mutant plants was observed. In addition, the lesion areas of *etr2* and *etr3* were significantly greater than those of DJ, indicating that *OsETR2* and *OsETR3* function as positive regulators in ShB resistance and that guanine induces rice resistance to *R. solani* YWK196 in an *OsETR2*/*OsETR3*-dependent manner. Col-0, *etr1* and *ein2* mutants were injected with *Pst* DC3000 after 2 h of guanine and mock treatment. As a result, guanine failed to induce plant resistance to *Pst* DC3000 in the *etr1* and *ein2* mutants (Figure 4E), indicating that guanine activated plant immunity and was dependent on ethylene signaling in *Arabidopsis*.

**FIGURE 5:**
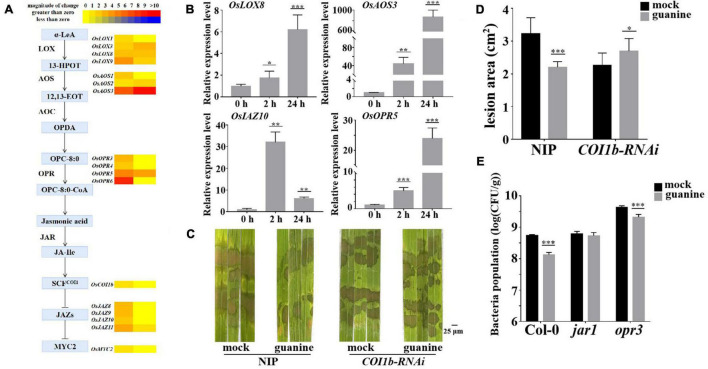
Guanine upregulated the expression of JA signaling pathway genes and enhanced plant disease resistance dependent on the JA signaling pathway. **(A)** Heatmap of JA-related gene expression after guanine treatment. **(B)** qRT-PCR for the detection of JA pathway genes *OsLOX8*, *OsAOS3*, *OsOPR5*, and *OsJAZ10.* The horizontal coordinates indicate the time of guanine- and mock-treated rice, and the vertical coordinates indicate the relative gene expression levels of guanine compared to the mock at the same time point. **(C)** Evaluation of the effect of guanine on *COI1b-RNAi* against *R*. *solani.* **(D)** Measurement of the lesion area by ImageJ. Lesion area after 3 days of inoculation with *R. solani* strain YWK196 after guanine treatment in NIP and *COI1b-RNAi*. Asterisks indicate *P* < 0.001 (***) and *P* < 0.05 (*) in Student’s *t*-test analysis. **(E)** Inoculation of *Pst* DC3000 after guanine treatment and bacterial growth in Col-0, *jar1* and *opr3* after 3 days. Asterisks indicate *P* < 0.001 (***) in Student’s *t*-test analysis.

In addition, both RNA-seq and qRT-PCR showed that JA biosynthesis- and signal transduction-related gene expression were upregulated by guanine, as illustrated in Figures 5A,B. To explore whether JA signaling plays a role in the guanine-mediated resistance response, *OsCOI1b-RNAi* and NIP (wild type) were inoculated with *R. solani* that was treated with guanine and mock 2 h later. The results showed that guanine failed to reduce the lesion area in *COI1b-RNAi* plants (Figures 5C,D), suggesting that the JA receptor *OsCOI1b* was necessary for guanine-induced rice resistance to ShB. Furthermore, a *Pst* DC3000 inoculation experiment was conducted in a JA biosynthesis mutant in *Arabidopsis.* As shown in Figure 5E, guanine treatment did not reduce the bacterial population in *jar1* mutant plants but significantly reduced the bacterial population in *opr3*. This difference indicated that guanine-induced resistance to *Pst* DC3000 is dependent on *AtJAR1* but not *AtOPR3*. Taken together, these results suggested that JA signaling is essential for guanine-induced plant resistance.

**FIGURE 6:**
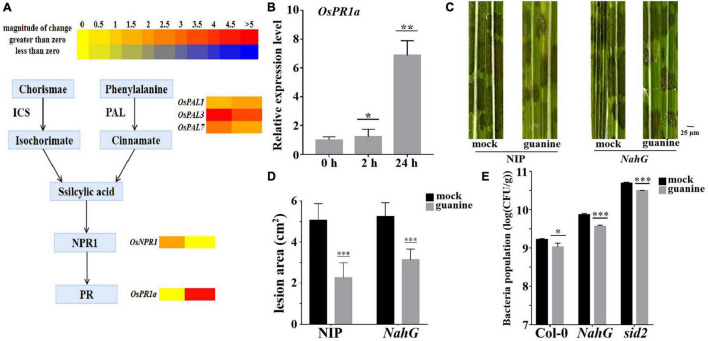
Guanine upregulated the expression of SA signaling pathway genes but still enhanced plant disease resistance in SA-deficient mutant plants. **(A)** Heatmap analysis of SA biosynthesis and responsive gene expression after guanine treatment at 2 and 24 hpt. **(B)** qRT-PCR for the detection of the SA pathway gene *OsPR1a.* The horizontal coordinates indicate the time of guanine- and mock-treated rice, and the vertical coordinates indicate the relative gene expression levels of guanine compared to the mock at the same time point. Asterisks indicate *P* < 0.05 (*), *P* < 0.01 (**) in Student’s *t*-test analysis. **(C)** Disease symptoms of the *NahG* transgenic plants. Leaves of the rice plants treated with the mock and 100 ng/mL guanine prior to *R. solani* strain YWK196 inoculation. The disease symptoms were photographed at 3 dpi. **(D)** Measurement of the lesion area of **(C)** by ImageJ. Asterisks indicate *P* < 0.001 (***) in Student’s *t*-test analysis. **(E)** Bacterial population in Col-0, *NahG* and *sid2* plant leaves. *Arabidopsis* leaves treated with the mock and 100 ng/mL guanine prior to *Pst* DC3000 inoculation. Bacterial population was calculated at 3 dpi. Asterisks indicate *P* < 0.05 (*), *P* < 0.001 (***) in Student’s *t*-test analysis.

A heatmap was used to determine the role of SA in guanine-mediated defense responses, and the results showed that *OsPAL1*, *OsPAL3*, and *OsPAL7* (Tonnessen et al., 2015), which are involved in SA biosynthesis, were upregulated by guanine treatment. In addition, *OsNPR1* and *OsPR1a*, marker genes of the SA pathway (Sohn et al., 2007; Yuan et al., 2007), were significantly upregulated by guanine treatment (Figure 6A). Furthermore, qRT-PCR analysis showed similar results as the transcriptome data (Figure 6B). Then, SA-deficient *NahG* transgenic plants were used for the following experiments. Guanine significantly reduced the lesion area in both NIP and *NahG* plants (Figures 6C,D). Similarly, guanine enhanced *Arabidopsis* resistance to *Pst* DC3000 in *NahG* and *sid2* mutant plants (Figure 6E). These results suggested that SA signaling is not necessary for guanine-induced resistance to pathogens in plants.

## Discussion

**FIGURE 7:**
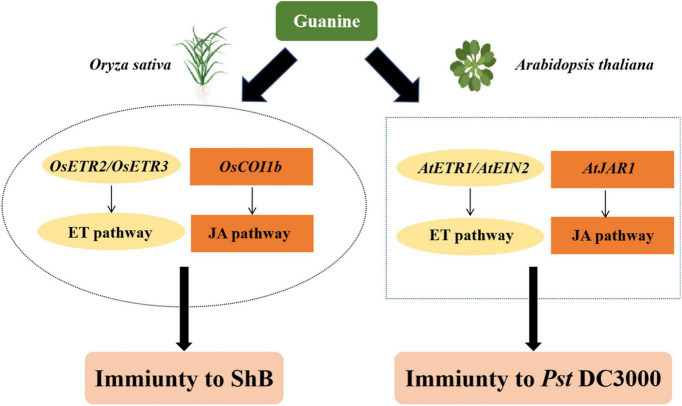
A proposed model of guanine-mediated plant immunity. In rice, *OsETR2*, *OsETR*3, and *OsCOI1b* are required for guanine-induced plant immunity to ShB. Similarly, guanine also needs *AtETR1*, *AtEIN2*, and *AtJAR1* to enhance plant resistance to *pst* DC3000 in *Arabidopsis.*

Here, we proposed a possible model to summarize the function of guanine in triggering plant immunity based on the above results (Figure 7). Guanine-enhanced rice resistance to ShB depends on the ET receptors *OsETR2* and *OsETR3* and the JA receptor *OsCOI1b*. In *Arabidopsis*, guanine-induced plant resistance to *Pst* DC3000 required *AtJAR1*, *AtETR1*, and *AtEIN2*. In summary, guanine activated jasmonic acid and ethylene signaling pathways to protect plants against pathogens. The mechanism of rice resistance to ShB is still largely unknown, and ShB control relies heavily on chemical fungicides (Zheng et al., 2013; Li et al., 2019; Wang et al., 2021). In this study, 100 ng/mL guanine significantly enhanced rice resistance to ShB, providing a novel, green and efficient strategy for ShB prevention and control.

At present, biological agents are usually used for the prevention and treatment of ShB in agricultural production. Rice ShB was effectively controlled by 0.1 mg/L Cu-based water-dispersible humic acid (Cu-WH) fungicide (Tang et al., 2019). In addition, previous studies have demonstrated that extracts of *Ginkgo biloba* outer seedcoats can inhibit the growth of *R. solani* Kuhn AG-1 and are considered most effective at a concentration of 250 μg/mL (Oh et al., 2015). Similarly, the rhizobacterium *Bacillus amyloliquefaciens* (SN13), as a biocontrol agent, can enhance immune responses against *R. solani* in rice by modulating various physiological, metabolic and molecular functions (Srivastava et al., 2016). In this study, 100 ng/mL guanine significantly enhanced the resistance of rice to ShB in both greenhouses and fields by triggering rice immunity (Figure 1). Furthermore, the concentration of guanine (100 ng/mL) also activated the ROS burst, MAPK phosphorylation, callose deposition and the expression of pathogen resistance-associated genes (Figures 2, 3), which was similar to the effects of Cu^2+^ and flg22. In the field, *R. solani* was inoculated on rice after 2 h of guanine treatment. The guanine-treated rice maintained better resistance to ShB after 3–5 days than the mock rice (Figures 1C,D). This finding indicates that the resistance of guanine-induced plants to ShB can be maintained for at least 3–5 days in the field. Thus, guanine is a highly efficient and environmentally friendly plant elicitor for ShB prevention and control in agricultural plant production.

In general, JA- and ET-mediated signaling pathways are considered to play a synergistic role in defense responses to necrotizing pathogens such as *R. solani* (Glazebrook, 2005). The JA/ET signaling pathway plays a crucial role in WRKY4-mediated defense responses to rice sheath blight (Wang et al., 2015). ET also plays an important role in plant growth and development (van Loon et al., 2006). ET is synthesized from the precursor intermediate 1-aminocyclopropane-1-carboxylate (ACC), which is catalyzed by ACC oxidase and derived from S-adenosylmethionine (SAM) *via* a reaction catalyzed by the ACC synthase ACS (Bleecker and Kende, 2000). A previous study showed that ET plays an essential role in rice immunity. For example, exogenous ET application induces pathogenesis-related (PR) gene expression in rice (Agrawal et al., 2001). In addition, overexpression of the ET biosynthetic gene *OsACS2* can enhance rice resistance to ShB (Helliwell et al., 2013). These studies indicate that the ET pathway can positively regulate rice resistance to ShB. Similarly, our results confirmed that guanine regulates rice disease resistance through the ET pathway. Transcriptome analysis showed that guanine treatment increased the upregulated expression of the ET biosynthesis genes *OsSAMS1* and *OsACO7* and ET response transcription factors *OsERF62* (Figures 4A,B). Interestingly, the ET receptors *OsETR2* and *OsETR3* were more sensitive to *R. solani* than the wild-type plants, and guanine treatment did not increase plant resistance to *R. solani* (Figures 4C,D). A possible explanation for this might be that guanine-induced resistance to ShB mainly depends on ET signal transduction. In *Arabidopsis*, guanine failed to induce plant resistance to *Pst* DC3000 in the *etr1* and *ein2* mutants (Figure 4E). These results suggested that guanine-mediated resistance to *Pst* DC3000 depends on ET signaling.

On the other hand, jasmonic acid plays a crucial role in plant resistance to insect and fungal pathogens. Exogenous JA can activate the expression of hundreds of defense-related genes, and the resulting feedback leads to the upregulation of the biosynthesis of resistance proteins and metabolites, including antifungal proteins and plant antitoxins (Ferrari et al., 2003; Srivastava et al., 2018; Ruan et al., 2019; Hu et al., 2021). In addition, *WRKY30* overexpression in rice enhances resistance to the rice sheath blight fungus *R. solani*, which also depends on the JA pathway (Peng et al., 2012). This study found that guanine was involved in the induction of JA biosynthesis and responses in plants, which was largely dependent on the receptor *OsCOI1b* for the regulation of rice resistance to ShB. Furthermore, guanine-enhanced plant resistance to pathogens also required *AtJAR1* but not the JA biosynthesis gene *AtOPR3* in *Arabidopsis* (Figure 5). Hence, we proposed that guanine-enhanced resistance to pathogens mainly depends on the ethylene and jasmonic acid signaling pathways.

## Conclusion

Our results demonstrated that guanine enhances the resistance of plants against bacteria and fungi. The ET and JA phytohormone signaling pathways are involved in guanine-mediated immunity. ET receptors *OsETR2* and *OsETR3* and JA receptor *OsCOI1b* are necessary for guanine-enhanced rice resistance to ShB. In *Arabidopsis*, guanine-induced plant resistance to *Pst* DC3000 depends on *AtJAR1*, *AtETR1*, and *AtEIN2*.

## Data Availability Statement

The original contributions presented in the study are publicly available. This data can be found here: National Center for Biotechnology Information (NCBI) BioProject database under accession number PRJNA767282.

## Author Contributions

XD designed the research and wrote the manuscript. LW performed the experiments, analyzed the data and wrote the manuscript. HL performed experiments and wrote the manuscript. CL and QW performed experiments. ZY and YL wrote the manuscript. All authors have read and agreed to the published version of the manuscript.

## Conflict of Interest

QW is employed by Shandong Pengbo Biotechnology Co., Ltd., Tai’an, Shandong, China. The remaining authors declare that the research was conducted in the absence of any commercial or financial relationships that could be construed as a potential conflict of interest.

## Publisher’s Note

All claims expressed in this article are solely those of the authors and do not necessarily represent those of their affiliated organizations, or those of the publisher, the editors and the reviewers. Any product that may be evaluated in this article, or claim that may be made by its manufacturer, is not guaranteed or endorsed by the publisher.

## Footnotes

1
https://imagej.nih.gov/ij/

2
https://www.genomics.cn/

3
http://bar.utoronto.ca/ntools/cgi-bin/ntools_heatmapper.cgi

## Supplementary Material

Supplementary Figure 1 Guanine enhanced *Arabidopsis* resistance to *Pst* DC3000. **(A)** Disease phenotype in Col-0 plants after treatment with 0, 1, 10, and 100 ng/mL guanine at 3 days. **(B)** Bacterial growth in Col-0 plants after treatment with 0, 1, 10, and 100 ng/mL guanine at 3 days (*n* = 6).

Supplementary Figure 2 Guanine failed to inhibit the growth of fungal and bacterial pathogens. **(A)** RS105 was grown in PSA medium containing 0, 10, and 100 ng/mL guanine. **(B)** PXO99A was grown in PSA medium containing 0, 10, and 100 ng/mL guanine. **(C)** YWK196 was grown in PDA medium containing 0, 1, 10, 100, and 200 ng/mL guanine. **(D)** *Pst* DC3000 was grown in PSA medium containing 0, 1, 10, 100, and 200 ng/mL guanine.

Supplementary Table 2 Quantitative metabolomic analysis and overall metabolite identification based on HPLC-LC/MS with ZNC.
