# Supplementary figures and images for: A Novel Guanine Elicitor Stimulates Immunity in Arabidopsis and Rice by Ethylene and Jasmonic Acid Signaling Pathways
Source: Front Plant Sci. 2022 Feb 17;13:841228. doi: 10.3389/fpls.2022.841228 (PMC8893958; doi:10.3389/fpls.2022.841228)

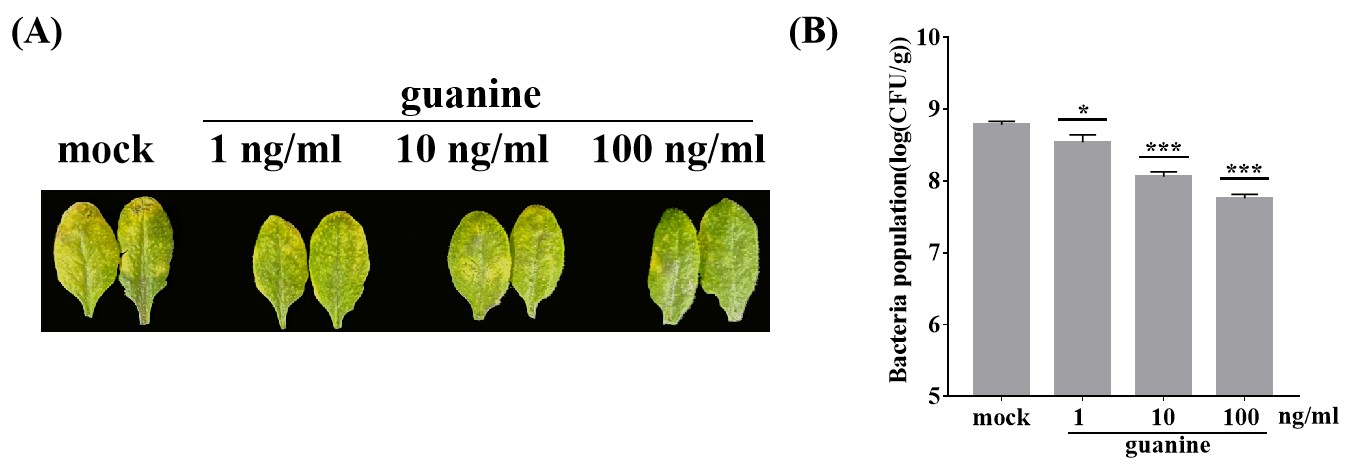

Supplement: Supplementary Figure 1 — Guanine enhanced Arabidopsis resistance to Pst DC3000. (A) Disease phenotype in Col-0 plants after treatment with 0, 1, 10, and 100 ng/mL guanine at 3 days. (B) Bacterial growth in Col-0 plants after treatment with 0, 1, 10, and 100 ng/mL guanine at 3 days (n = 6). [file Image_1.JPEG]

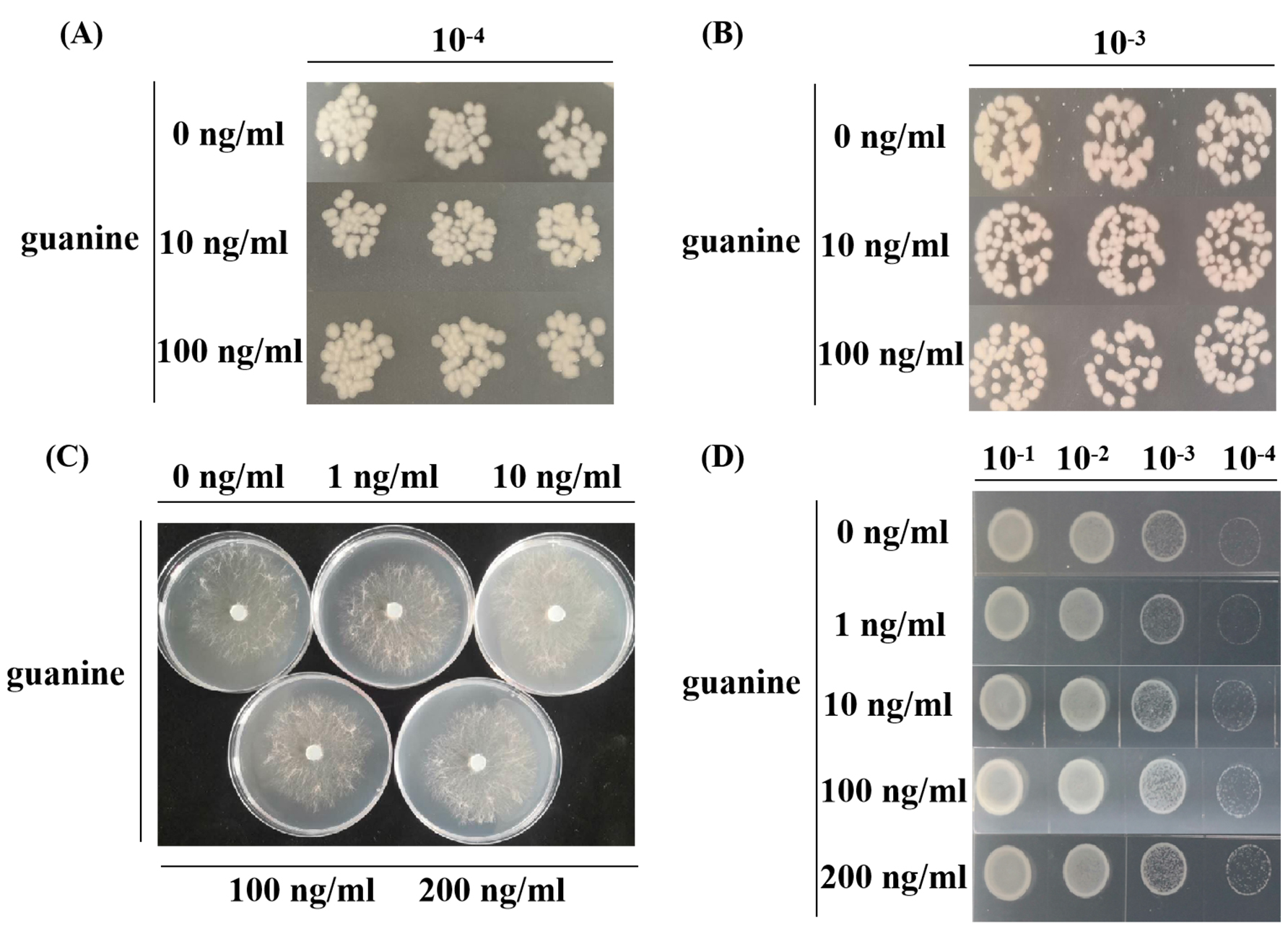

Supplement: Supplementary Figure 2 — Guanine failed to inhibit the growth of fungal and bacterial pathogens. (A) RS105 was grown in PSA medium containing 0, 10, and 100 ng/mL guanine. (B) PXO99A was grown in PSA medium containing 0, 10, and 100 ng/mL guanine. (C) YWK196 was grown in PDA medium containing 0, 1, 10, 100, and 200 ng/mL guanine. (D) Pst DC3000 was grown in PSA medium containing 0, 1, 10, 100, and 200 ng/mL guanine. [file Image_2.jpeg]
