# Supplementary material for: A Novel Guanine Elicitor Stimulates Immunity in Arabidopsis and Rice by Ethylene and Jasmonic Acid Signaling Pathways
Source: Front Plant Sci. 2022 Feb 17;13:841228. doi: 10.3389/fpls.2022.841228 (PMC8893958; doi:10.3389/fpls.2022.841228)
Supplement: Supplementary Table 2 — Quantitative metabolomic analysis and overall metabolite identification based on HPLC-LC/MS with ZNC. [file Table_1.docx]

**[Supplementary](https://www.mdpi.com/1422-0067/21/21/7974/htm#app1-ijms-21-07974) Table 1: List of qRT-PCR primers used in this study**

Primers Sequence shown in 5’→3’ orientation

qPCR-OsAOX1c-F GCGTCGGATGTTCATTTTCA

qPCR-OsAOX1c-R CCGCCGTAAGATTTCTTCAGT

qPCR-OsROBHE-F GCAGAACAGAACGATCCACCA

qPCR-OsROBHE-R CCCAGTAGAAGTGTGCCCTG

qPCR-OsACO7-F ACTCGCTCATCGGAAGCAAA

qPCR-OsACO7-R GCCATGGTTTTCCACCCAGA

qPCR-OsAOS3-F CGGGCGTCGTCGGGATAG

qPCR-OsAOS3-R AACATCCACCAACCTAGTACAGTAC

qPCR-OsPR1a-F CGTCTTCATCACCTGCAACTACTC

qPCR-OsPR1a-R CATGCATAAACACGTAGCATAGCA

qPCR-OsSAMS1-F ATCATACGCCATCGGTGTCC

qPCR-OsSAMS1-R TTCCGAAGTGACCGTAAGCC

qPCR-OsWRKY53-F GTTCATCACGTCGTTCACCG

qPCR-OsWRKY53-R GTCGGGGACGCCAAGATATG

qPCR-OsOPR5-F ACAACTCGGTTGCTGATGCT

qPCR-OsOPR5-R TGTGGTGTGAATACATCTGCGT

qPCR-OsERF62-F GAGCTTCGACGACTTCCCAA

qPCR-OsERF62-R GACCAGCAGTACGAAGACCC

qPCR-OsJAZ10-F AAGCCGCGTTTTGTTTCTCG

qPCR-OsJAZ10-R TCCTCGAAGACGACCATCCT

qPCR-OsActin-F TGTATGCCAGTGGTCGTACCA

qPCR-OsActin-R CCAGCAAGGTCGAGACGAA

**[Supplementary](https://www.mdpi.com/1422-0067/21/21/7974/htm#app1-ijms-21-07974) Table 2: The analysis of ZNC components**

|  | Substance | Substance Category | 1 | |
| --- | --- | --- | --- | --- |
| Lipids | "14,15-Dehydrocrepenynic acid" | Lipids | 4720000 | |
| Lipids | "9-Hydroxy-(10E,12Z,15Z)-octadecatrienoic acid" | Lipids | 29500000 | |
| Lipids | delta-Tridecalactone | Lipids | 74600 | |
| Lipids | MAG (18:4) isomer1 | Lipids | 1400000 | |
| Lipids | DGMG (18:2) isomer2 | Lipids | 9 | |
| Lipids | MAG (18:3) isomer5 | Lipids | 31300 | |
| Lipids | DGMG (18:2) isomer1 | Lipids | 7340 | |
| Lipids | DGMG (18:2) isomer3 | Lipids | 5200 | |
| Lipids | MAG (18:2) isomer1 | Lipids | 13200 | |
| Lipids | MAG (18:4) isomer2 | Lipids | 9 | |
| Lipids | MAG (18:1) isomer2 | Lipids | 343000 | |
| Lipids | LysoPC 16:1 (2n isomer) | Lipids | 9 | |
| Lipids | LysoPC 18:2 | Lipids | 9 | |
| Lipids | LysoPC 18:3 | Lipids | 188000 | |
| Lipids | LysoPC 16:0 | Lipids | 527000 | |
| Lipids | LysoPE 18:1 (2n isomer) | Lipids | 9 | |
| Lipids | LysoPC 18:1 (2n isomer) | Lipids | 100000 | |
| Lipids | LysoPC 16:2 (2n isomer) | Lipids | 9 | |
| Lipids | LysoPE 14:0 | Lipids | 9 | |
| Lipids | LysoPC 18:3 (2n isomer) | Lipids | 489000 | |
| Lipids | LysoPC 14:0 | Lipids | 9 | |
| Lipids | LysoPC 18:2 (2n isomer) | Lipids | 41700 | |
| Lipids | LysoPE 18:2 (2n isomer) | Lipids | 112000 | |
| Lipids | LysoPE 16:0 | Lipids | 148000 | |
| Lipids | LysoPC 18:1 (2n isomer) | Lipids | 9 | |
| Lipids | LysoPE 18:0 | Lipids | 41500 | |
| Lipids | Punicic acid | Lipids | 11800000 | |
| Lipids | MAG (18:2) | Lipids | 9190 | |
| Lipids | MAG (18:3) isomer3 | Lipids | 366000 | |
| Lipids | MGMG (18:2) isomer1 | Lipids | 6990 | |
| Lipids | "Octadeca-11E,13E,15Z-trienoic acid" | Lipids | 98300000 | |
| Lipids | MAG (18:3) isomer4 | Lipids | 290000 | |
| Lipids | 4-Hydroxysphinganine | Lipids | 1420000 | |
| Lipids | DGMG (18:1) | Lipids | 7840 | |
| Lipids | LysoPC 15:1 | Lipids | 9 | |
| Lipids | LysoPC 15:0 | Lipids | 9 | |
| Lipids | MAG (18:3) isomer2 | Lipids | 24000 | |
| Lipids | MGMG (18:2) isomer2 | Lipids | 9 | |
| Lipids | LysoPC 18:0 (2n isomer) | Lipids | 9 | |
| Lipids | LysoPC 17:0 | Lipids | 9 | |
| Lipids | MAG (18:3) isomer1 | Lipids | 32600 | |
| Lipids | α-Linolenic acid | Lipids | 9 | |
| Lipids | "8,15-DiHETE" | Lipids | 88700 | |
| Lipids | Lauric acid (C12:0) | Lipids | 10200 | |
| Lipids | Myristoleic acid (C14:1) | Lipids | 3260 | |
| Lipids | "9,10-EODE" | Lipids | 44700000 | |
| Lipids | 9-HOTrE | Lipids | 249000 | |
| Lipids | 13-KODE | Lipids | 291000 | |
| Lipids | 13-HpOTrE(r) | Lipids | 9880000 | |
| Lipids | 9-HpOTrE | Lipids | 6050000 | |
| Lipids | 13-HOTrE(r) | Lipids | 29900000 | |
| Lipids | "12,13-EODE" | Lipids | 185000 | |
| Lipids | 13-HPODE | Lipids | 7390000 | |
| Lipids | LysoPE 18:3 | Lipids | 9 | |
| Lipids | LysoPE 18:1 (2n isomer) | Lipids | 9 | |
| Lipids | LysoPC 20:4 | Lipids | 9 | |
| Lipids | LysoPC 14:0 (2n isomer) | Lipids | 9 | |
| Lipids | LysoPC 16:0 (2n isomer) | Lipids | 512000 | |
| Lipids | LysoPC 18:0 | Lipids | 9 | |
| Lipids | LysoPC 20:1 (2n isomer) | Lipids | 9 | |
| Lipids | LysoPC 20:1 | Lipids | 9 | |
| Lipids | LysoPE 16:0 (2n isomer) | Lipids | 29900 | |
| Lipids | Palmitaldehyde | Lipids | 12100000 | |
| Lipids | 1-Octadecanol | Lipids | 1180000 | |
| Lipids | 1-Eicosanol | Lipids | 2050000 | |
| Lipids | "1,18-Octadecanediol" | Lipids | 9 | |
| Lipids | Linoleic acid | Lipids | 163000 | |
| Lipids | cis-Gondoic acid | Lipids | 5870000 | |
| Organic acids and derivatives | Xanthurenic acid | Organic acids and derivatives | 112000 | |
| Organic acids and derivatives | 5-Aminolevulinate | Organic acids and derivatives | 130000000 | |
| Organic acids and derivatives | Phosphoric acid | Organic acids and derivatives | 25600000 | |
| Organic acids and derivatives | 10-Formyl-THF | Organic acids and derivatives | 3000000 | |
| Organic acids and derivatives | Anthranilate O-hexosyl-O-hexoside | Organic acids and derivatives | 7130000 | |
| Organic acids and derivatives | Argininosuccinate | Organic acids and derivatives | 392000 | |
| Organic acids and derivatives | Kynurenic acid O-hexside | Organic acids and derivatives | 53600 | |
| Organic acids and derivatives | Gallic acid O-Hexoside | Organic acids and derivatives | 9 | |
| Organic acids and derivatives | Diethyl phosphate | Organic acids and derivatives | 1360000 | |
| Organic acids and derivatives | 2-Isopropylmalate | Organic acids and derivatives | 75600 | |
| Organic acids and derivatives | Syringic acid O-glucoside | Organic acids and derivatives | 9 | |
| Organic acids and derivatives | 2-Aminoethanesulfonic acid | Organic acids and derivatives | 663000 | |
| Organic acids and derivatives | 2-Aminoethanesulfinic acid | Organic acids and derivatives | 376000 | |
| Organic acids and derivatives | 3-Hydroxybutyrate | Organic acids and derivatives | 9 | |
| Organic acids and derivatives | Kynurenic acid O-hexside | Organic acids and derivatives | 5440000 | |
| Organic acids and derivatives | "Ethyl 3,4-Dihydroxybenzoate (Ethyl protocatechuate)" | Organic acids and derivatives | 184000 | |
| Organic acids and derivatives | Benzoic acid | Organic acids and derivatives | 9230000 | |
| Organic acids and derivatives | Glutaric acid | Organic acids and derivatives | 5370000 | |
| Organic acids and derivatives | Adipic acid | Organic acids and derivatives | 1110000 | |
| Organic acids and derivatives | Azelaic acid | Organic acids and derivatives | 3910000 | |
| Organic acids and derivatives | Sebacate | Organic acids and derivatives | 119000 | |
| Organic acids and derivatives | 2-Methylsuccinic acid | Organic acids and derivatives | 4390000 | |
| Organic acids and derivatives | Benzoylformic acid | Organic acids and derivatives | 9 | |
| Organic acids and derivatives | 6-Aminocaproic acid | Organic acids and derivatives | 201000000 | |
| Organic acids and derivatives | 4-Oxopentanoate | Organic acids and derivatives | 110000 | |
| Organic acids and derivatives | Terephthalic acid | Organic acids and derivatives | 8870000 | |
| Organic acids and derivatives | Phthalic acid | Organic acids and derivatives | 854000 | |
| Organic acids and derivatives | 4-Guanidinobutyric acid | Organic acids and derivatives | 69900000 | |
| Organic acids and derivatives | 2-Hydroxyisocaproic acid | Organic acids and derivatives | 1240000 | |
| Organic acids and derivatives | Dl-2-Aminooctanoic acid | Organic acids and derivatives | 9 | |
| Organic acids and derivatives | 4-Acetamidobutyric acid | Organic acids and derivatives | 5120000 | |
| Organic acids and derivatives | Methyl gallate | Organic acids and derivatives | 20300 | |
| Organic acids and derivatives | Ethyl gallate | Organic acids and derivatives | 9 | |
| Organic acids and derivatives | Vanillin | Organic acids and derivatives | 438000 | |
| Organic acids and derivatives | Methylmalonic acid | Organic acids and derivatives | 27400000 | |
| Organic acids and derivatives | Gallic acid O-Hexoside | Organic acids and derivatives | 9 | |
| Organic acids and derivatives | D-Pantothenic dcid | Organic acids and derivatives | 405000 | |
| Organic acids and derivatives | 3-Hydroxy-3-methyl butyric acid | Organic acids and derivatives | 2020000 | |
| Organic acids and derivatives | Methyl benzoate | Organic acids and derivatives | 108000 | |
| Organic acids and derivatives | D-Erythronolactone | Organic acids and derivatives | 160000 | |
| Organic acids and derivatives | p–Hydroxyphenyl acetic acid | Organic acids and derivatives | 739000 | |
| Organic acids and derivatives | Succinic acid | Organic acids and derivatives | 88800000 | |
| Organic acids and derivatives | Suberic acid | Organic acids and derivatives | 340000 | |
| Organic acids and derivatives | L(-)-Malic acid | Organic acids and derivatives | 89500000 | |
| Organic acids and derivatives | 2-Hydroxybutanoic acid | Organic acids and derivatives | 2580000 | |
| Organic acids and derivatives | Citric acid | Organic acids and derivatives | 41700000 | |
| Organic acids and derivatives | (S)-(-)-2-Hydroxyisocaproic acid | Organic acids and derivatives | 9290000 | |
| Organic acids and derivatives | Fumaric acid | Organic acids and derivatives | 750000 | |
| Organic acids and derivatives | Citraconic acid | Organic acids and derivatives | 4620000 | |
| Organic acids and derivatives | 3-Aminosalicylic acid | Organic acids and derivatives | 278000 | |
| Organic acids and derivatives | Dodecanedioic aicd | Organic acids and derivatives | 140000 | |
| Organic acids and derivatives | A-Ketoglutaric acid | Organic acids and derivatives | 106000 | |
| Organic acids and derivatives | α-Hydroxyisobutyric acid | Organic acids and derivatives | 622000 | |
| Organic acids and derivatives | Cis-Aconitic acid | Organic acids and derivatives | 9 | |
| Organic acids and derivatives | "3,4-Dihydroxybenzeneacetic acid" | Organic acids and derivatives | 251000 | |
| Organic acids and derivatives | 3-Hydroxypropanoic acid | Organic acids and derivatives | 383000 | |
| Organic acids and derivatives | 3-Hydroxyanthranilic acid | Organic acids and derivatives | 9 | |
| Organic acids and derivatives | 4-Hydroxybenzaldehyde | Organic acids and derivatives | 11500000 | |
| Organic acids and derivatives | "2,3-Dihydroxybenzoic acid" | Organic acids and derivatives | 9 | |
| Organic acids and derivatives | 2-Hydroxy-2-methyl butyric acid | Organic acids and derivatives | 9 | |
| Organic acids and derivatives | 4-Hydroxy-2-oxoglutaric acid | Organic acids and derivatives | 9 | |
| Organic acids and derivatives | 4-Hydroxybenzoic acid | Organic acids and derivatives | 17500000 | |
| Organic acids and derivatives | "N,N-Dimethylformamide" | Organic acids and derivatives | 271000000 | |
| Organic acids and derivatives | trans-Citridic acid | Organic acids and derivatives | 2330000 | |
| Organic acids and derivatives | γ-aminobutyric acid | Organic acids and derivatives | 16800000 | |
| Organic acids and derivatives | ethylmalonate | Organic acids and derivatives | 193000 | |
| Organic acids and derivatives | 2-(Formylamino)benzoic acid | Organic acids and derivatives | 11600000 | |
| Organic acids and derivatives | Aminomalonic acid | Organic acids and derivatives | 13400000 | |
| Organic acids and derivatives | Ureidoisobutyric acid | Organic acids and derivatives | 563000 | |
| Organic acids and derivatives | (Rs)-Mevalonic acid | Organic acids and derivatives | 3130000 | |
| Organic acids and derivatives | 2-Methylglutaric acid | Organic acids and derivatives | 601000 | |
| Organic acids and derivatives | 5-hydroxyhexanoic acid | Organic acids and derivatives | 758000 | |
| Organic acids and derivatives | "Eudesmic acid (3,4,5-trimethoxybenzoic acid)" | Organic acids and derivatives | 9 | |
| Organic acids and derivatives | Anthranilic acid | Organic acids and derivatives | 3010000 | |
| Organic acids and derivatives | D-Xylonic acid | Organic acids and derivatives | 9180000 | |
| Organic acids and derivatives | Oxalic acid | Organic acids and derivatives | 3570000 | |
| Organic acids and derivatives | Coumalic acid | Organic acids and derivatives | 9 | |
| Organic acids and derivatives | Phosphoenolpyruvate trisodium salt | Organic acids and derivatives | 9 | |
| Organic acids and derivatives | D-Galacturonic acid | Organic acids and derivatives | 1590000 | |
| Organic acids and derivatives | Benzyl acetate | Organic acids and derivatives | 118000 | |
| Carbohydrates | D(-)-Threose | Carbohydrates | 540000 | |
| Carbohydrates | Ribulose-5-phosphate | Carbohydrates | 153000 | |
| Carbohydrates | Glucosamine | Carbohydrates | 9 | |
| Carbohydrates | Maltotetraose | Carbohydrates | 307000 | |
| Carbohydrates | Trehalose 6-phosphate | Carbohydrates | 59500 | |
| Carbohydrates | D-(+)-Sucrose | Carbohydrates | 2970000 | |
| Carbohydrates | D(+)-Melezitose | Carbohydrates | 9 | |
| Carbohydrates | "D-(+)-Glucono-1,5-lactone" | Carbohydrates | 427000 | |
| Carbohydrates | D(+)-Glucose | Carbohydrates | 2910000 | |
| Carbohydrates | DL-Arabinose | Carbohydrates | 1990000 | |
| L-Fucose | L-Fucose | Carbohydrates | 906000 | |
| Carbohydrates | N-Acetyl-D-glucosamine | Carbohydrates | 26000000 | |
| Carbohydrates | D-Glucose 6-phosphate | Carbohydrates | 3730000 | |
| Carbohydrates | D-Fructose 6-phosphate-disodium salt | Carbohydrates | 1650000 | |
| Carbohydrates | Galactinol | Carbohydrates | 4290000 | |
| Carbohydrates | Glucose-1-phosphate | Carbohydrates | 3710000 | |
| Carbohydrates | D-(+)-Mannose | Carbohydrates | 9730000 | |
| Carbohydrates | D-(+)-Galactose | Carbohydrates | 5400000 | |
| Carbohydrates | D-Fructose 6-phosphate-disodium salt | Carbohydrates | 4280000 | |
| Carbohydrates | Galactinol Dihydrate | Carbohydrates | 4550 | |
| Carbohydrates | Melibiose | Carbohydrates | 1120000 | |
| Carbohydrates | Carbohydrates | Carbohydrates | 278000 | |
| Others | N-Lauryldiethanolamine | Others | 1800000 | |
| Others | 2-Deoxyribose 1-phosphate | Others | 1510000 | |
| Others | 2-Deoxyribose 5'-phosphate | Others | 821000 | |
| Others | N-Acetylglucosamine 1-phosphate | Others | 263000 | |
| Others | Gluconic acid | Others | 52300000 | |
| Others | L-Gulonic-γ-lactone | Others | 548000 | |
| Others | D-glucoronic acid | Others | 809000 | |
| Others | D-Glucose-6-phosphate disodium salt | Others | 6340000 | |
| Nucleotide and derivates | N2-methylguanosin | Nucleotide and derivates | 9 | |
| Nucleotide and derivates | Adenosine 3'-monophosphate | Nucleotide and derivates | 4460000 | |
| Nucleotide and derivates | Nicotinic acid adenine dinucleotide | Nucleotide and derivates | 86200 | |
| Nucleotide and derivates | Inosine 5'-monophosphate | Nucleotide and derivates | 585000 | |
| Nucleotide and derivates | iP7G | Nucleotide and derivates | 2220000 | |
| Nucleotide and derivates | Adenosine 5'-monophosphate | Nucleotide and derivates | 8610000 | |
| Nucleotide and derivates | Guanosine 5'-monophosphate | Nucleotide and derivates | 1570000 | |
| Nucleotide and derivates | Cyclic AMP | Nucleotide and derivates | 1470000 | |
| Nucleotide and derivates | Uridine 5'-diphospho-D-glucose | Nucleotide and derivates | 77700 | |
| Nucleotide and derivates | 2'-Deoxyinosine | Nucleotide and derivates | 1700000 | |
| Nucleotide and derivates | 2'-Deoxyinosine-5'-monophosphate | Nucleotide and derivates | 75600 | |
| Nucleotide and derivates | 6-Methylmercaptopurine | Nucleotide and derivates | 144000000 | |
| Nucleotide and derivates | Adenosine O-ribose | Nucleotide and derivates | 440000 | |
| Nucleotide and derivates | Succinyladenosine | Nucleotide and derivates | 82300000 | |
| Nucleotide and derivates | Purine | Nucleotide and derivates | 131000 | |
| Nucleotide and derivates | Thymine | Nucleotide and derivates | 925000 | |
| Nucleotide and derivates | Nucleotide and derivates | Nucleotide and derivates | 3120000 | |
| Nucleotide and derivates | Cytosine | Nucleotide and derivates | 28800000 | |
| Nucleotide and derivates | Adenine | Nucleotide and derivates | 48400000 | |
| Nucleotide and derivates | 5-Methylcytosine | Nucleotide and derivates | 2780000 | |
| Nucleotide and derivates | 1-Methylxanthine | Nucleotide and derivates | 9 | |
| Nucleotide and derivates | 2-Hydroxy-6-aminopurine | Nucleotide and derivates | 18300000 | |
| Nucleotide and derivates | Adenosine | Nucleotide and derivates | 720000000 | |
| Nucleotide and derivates | Xanthine | Nucleotide and derivates | 23900000 | |
| Nucleotide and derivates | Uracil | Nucleotide and derivates | 6430000 | |
| Nucleotide and derivates | 5-Methyluridine | Nucleotide and derivates | 1910000 | |
| Nucleotide and derivates | Thymidine | Nucleotide and derivates | 7660000 | |
| Nucleotide and derivates | Uridine 5'-monophosphate | Nucleotide and derivates | 24400000 | |
| Nucleotide and derivates | Guanine | Nucleotide and derivates | 20100000 | |
| Nucleotide and derivates | Inosine | Nucleotide and derivates | 10200000 | |
| Nucleotide and derivates | Guanosine | Nucleotide and derivates | 281000000 | |
| Nucleotide and derivates | Deoxyguanosine | Nucleotide and derivates | 91600000 | |
| Nucleotide and derivates | Deoxycytidine | Nucleotide and derivates | 57900000 | |
| Nucleotide and derivates | 3-Methylxanthine | Nucleotide and derivates | 384000 | |
| Nucleotide and derivates | Xanthosine | Nucleotide and derivates | 96200000 | |
| Nucleotide and derivates | 8-Hydroxyguanosine | Nucleotide and derivates | 3110000 | |
| Nucleotide and derivates | 2'-Deoxycytidine-5'-monophosphate | Nucleotide and derivates | 832000 | |
| Nucleotide and derivates | 1-Methyladenosine | Nucleotide and derivates | 5300000 | |
| Nucleotide and derivates | 5'-Deoxy-5'-(methylthio)adenosine | Nucleotide and derivates | | 9 |
| Nucleotide and derivates | Guanosine monophosphate | Nucleotide and derivates | | 410000 |
| Nucleotide and derivates | "1,7-Dimethylxanthine" | Nucleotide and derivates | | 92100 |
| Nucleotide and derivates | 7-Methylxanthine | Nucleotide and derivates | | 9 |
| Nucleotide and derivates | 5-Hydroxymethyluracil | Nucleotide and derivates | | 9 |
| Nucleotide and derivates | Uridine 5’-diphosphate | Nucleotide and derivates | | 33500 |
| Nucleotide and derivates | 1-Methyladenine | Nucleotide and derivates | | 201000 |
| Nucleotide and derivates | Cytidine 5'-monophosphate (Cytidylic acid) | Nucleotide and derivates | | 9 |
| Nucleotide and derivates | 2'-Deoxyadenosine-5'-monophosphate | Nucleotide and derivates | | 6760000 |
| Nucleotide and derivates | Uridine 5'-monophosphate | Nucleotide and derivates | | 2860000 |
| Nucleotide and derivates | 1-methylguanidine | Nucleotide and derivates | | 347000 |
| Nucleotide and derivates | N6-Succinyl Adenosine | Nucleotide and derivates | | 65700000 |
| Nucleotide and derivates | 8-Hydroxy-2-deoxyguanosine | Nucleotide and derivates | | 562000 |
| Nucleotide and derivates | Cytidine | Nucleotide and derivates | | 87300000 |
| Nucleotide and derivates | Nucleotide and derivates | Nucleotide and derivates | | 9 |
| Nucleotide and derivates | 2'-Deoxyuridine | Nucleotide and derivates | | 603000 |
| Nucleotide and derivates | Deoxyadenosine | Nucleotide and derivates | | 310000000 |
| Nucleotide and derivates | 2-(dimethylamino)guanosine | Nucleotide and derivates | | 20500000 |
| Nucleotide and derivates | 7-methylguanine | Nucleotide and derivates | | 70600 |
| Nucleotide and derivates | β-Pseudouridine | Nucleotide and derivates | | 2130000 |
| Nucleotide and derivates | Hypoxanthine-9-β-D-arabinofuranoside | Nucleotide and derivates | | 37200000 |
| Nucleotide and derivates | Uridine 5'-diphosphoglucose disodium salt | Nucleotide and derivates | | 43500 |
| Nucleotide and derivates | UDP-α-D-glucose | Nucleotide and derivates | | 33200 |
| Nucleotide and derivates | Riboprine | Nucleotide and derivates | | 730000 |
| Nucleotide and derivates | Cordycepin | Nucleotide and derivates | | 7800000 |
| Phenolamides | N-Acetyl tryptamine | Phenolamides | | 282000 |
| Phenolamides | Spermidine | Phenolamides | | 9 |
| Phenolamides | Spermine | Phenolamides | | 141000000 |
| Phenolamides | "1,5-Diaminopentane" | Phenolamides | | 453000 |
| Phenolamides | Putrescine | Phenolamides | | 9 |
| Alcohols | Histidinol | Alcohols | | 30900000 |
| Alcohols | D-erythro-Dihydrosphingosine | Alcohols | | 51300 |
| Alcohols | D-Sorbitol | Alcohols | | 34400000 |
| Alcohols | Xylitol | Alcohols | | 2470000 |
| Alcohols | Inositol | Alcohols | | 882000 |
| Alcohols | D-Mannitol | Alcohols | | 7600000 |
| Alcohols | Dulcitol | Alcohols | | 44800000 |
| Alcohols | D-Arabitol | Alcohols | | 2620000 |
| Alcohols | "1,5-Anhydro-D-glucitol" | Alcohols | | 1010000 |
| Alcohols | Pantothenol | Alcohols | | 150000 |
| Alcohols | Mannitol | Alcohols | | 2850000 |
| Alcohols | 1-Decanol | Alcohols | | 50900000 |
| Alcohols | Meso-Erythritol | Alcohols | | 63200 |
| Phenylpropanoids | O-Feruloyl 4-hydroxylcoumarin | Phenylpropanoids | | 9 |
| Phenylpropanoids | Coumarin O-rutinoside | Phenylpropanoids | | 9 |
| Phenylpropanoids | trans-Cinnamate | Phenylpropanoids | | 1150000 |
| Phenylpropanoids | Homovanillic acid | Phenylpropanoids | | 455000 |
| Phenylpropanoids | 4-Methylumbelliferone | Phenylpropanoids | | 868000 |
| Phenylpropanoids | 3-(4-Hydroxyphenyl)propionic acid | Phenylpropanoids | | 994000 |
| Phenylpropanoids | 3-Hydroxy-4-methoxycinnamic acid | Phenylpropanoids | | 9 |
| Phenylpropanoids | "3,4,5-Trimethoxycinnamic acid" | Phenylpropanoids | | 128000 |
| Phenylpropanoids | p-Coumaric acid | Phenylpropanoids | | 790000 |
| Phenylpropanoids | Phenylpropanoids | Phenylpropanoids | | 520000 |
| Phenylpropanoids | 6-MethylCoumarin | Phenylpropanoids | | 9 |
| Phenylpropanoids | Coumarin | Phenylpropanoids | | 218000 |
| Phenylpropanoids | p-Coumaraldehyde | Phenylpropanoids | | 9 |
| Phenylpropanoids | 7-Methoxycoumarin | Phenylpropanoids | | 612000 |
| Phenylpropanoids | Acetosyringone | Phenylpropanoids | | 129000 |
| Phenylpropanoids | 4-Methoxycinna Maldehyde | Phenylpropanoids | | 593000 |
| Phenylpropanoids | Cinnamic acid | Phenylpropanoids | | 1000000 |
| Amino acid and derivatives | 3-Hydroxykynurenine | Amino acid and derivatives | | 668000 |
| Amino acid and derivatives | 2-Aminoadipic acid (L-Homoglutamic acid) | Amino acid and derivatives | | 2780000 |
| Amino acid and derivatives | Aspartic acid di-O-glucoside | Amino acid and derivatives | | 2980000 |
| Amino acid and derivatives | Lysine butyrate | Amino acid and derivatives | | 593000 |
| Amino acid and derivatives | L-Glutamine O-hexside | Amino acid and derivatives | | 511000 |
| Amino acid and derivatives | L-Glutamic acid O-glucoside | Amino acid and derivatives | | 672000 |
| Amino acid and derivatives | 3-(2-Naphthyl)-D-alanine | Amino acid and derivatives | | 471000 |
| Amino acid and derivatives | L-Homocitrulline | Amino acid and derivatives | | 2200000 |
| Amino acid and derivatives | L-Proline | Amino acid and derivatives | | 400000000 |
| Amino acid and derivatives | L-Citrulline | Amino acid and derivatives | | 4560000 |
| Amino acid and derivatives | L-Serine | Amino acid and derivatives | | 3540000 |
| Amino acid and derivatives | L-Aspartic acid | Amino acid and derivatives | | 10300000 |
| Amino acid and derivatives | L-Glutamic acid | Amino acid and derivatives | | 16300000 |
| Amino acid and derivatives | L-(-)-Cystine | Amino acid and derivatives | | 368000 |
| Amino acid and derivatives | L-Leucine | Amino acid and derivatives | | 8410000 |
| Amino acid and derivatives | L-Phenylalanine | Amino acid and derivatives | | 501000000 |
| Amino acid and derivatives | L-Threonine | Amino acid and derivatives | | 16400000 |
| Amino acid and derivatives | L-Kynurenine | Amino acid and derivatives | | 3010000 |
| Amino acid and derivatives | L-(+)-Lysine | Amino acid and derivatives | | 123000000 |
| Amino acid and derivatives | L-(-)-Tyrosine | Amino acid and derivatives | | 341000000 |
| Amino acid and derivatives | L-Histidine | Amino acid and derivatives | | 6260000 |
| Amino acid and derivatives | L-Valine | Amino acid and derivatives | | 438000000 |
| Amino acid and derivatives | L-(+)-Arginine | Amino acid and derivatives | | 119000000 |
| Amino acid and derivatives | "2,3-dimethylsuccinic acid" | Amino acid and derivatives | | 1020000 |
| Amino acid and derivatives | DL-Homocysteine | Amino acid and derivatives | | 3270000 |
| Amino acid and derivatives | Guanidineacetic acid | Amino acid and derivatives | | 388000 |
| Amino acid and derivatives | N-Acetyl-L-glutamic acid | Amino acid and derivatives | | 1390000 |
| Amino acid and derivatives | "(-)-3-(3,4-Dihydroxyphenyl)-2-methylalanine" | Amino acid and derivatives | | 811000 |
| Amino acid and derivatives | γ-Glu-Cys | Amino acid and derivatives | | 518000 |
| Amino acid and derivatives | 5-Aminovaleric acid | Amino acid and derivatives | | 820000 |
| Amino acid and derivatives | N6-Acetyl-L-lysine | Amino acid and derivatives | | 11900000 |
| Amino acid and derivatives | Glycyl-L-proline | Amino acid and derivatives | | 1220000 |
| Amino acid and derivatives | D-Alanyl-D-Alanine | Amino acid and derivatives | | 337000 |
| Amino acid and derivatives | Asp-phe | Amino acid and derivatives | | 1680000 |
| Amino acid and derivatives | Nα-Acetyl-L-glutamine | Amino acid and derivatives | | 10300000 |
| Amino acid and derivatives | L-Homoserine | Amino acid and derivatives | | 246000 |
| Amino acid and derivatives | Nα-Acetyl-L-arginine | Amino acid and derivatives | | 7670000 |
| Amino acid and derivatives | N-Isovaleroylglycine | Amino acid and derivatives | | 32500 |
| Amino acid and derivatives | N-Propionylglycine | Amino acid and derivatives | | 6060000 |
| Amino acid and derivatives | 1-Methylhistidine | Amino acid and derivatives | | 1370000 |
| Amino acid and derivatives | N-Glycyl-L-leucine | Amino acid and derivatives | | 14900000 |
| Amino acid and derivatives | L-Glutamine | Amino acid and derivatives | | 154000000 |
| Amino acid and derivatives | L-Cysteine | Amino acid and derivatives | | 748000 |
| Amino acid and derivatives | L-Asparagine | Amino acid and derivatives | | 2930000 |
| Amino acid and derivatives | N-(3-Indolylacetyl)-L-alanine | Amino acid and derivatives | | 95200 |
| Amino acid and derivatives | trans-4-Hydroxy-L-proline | Amino acid and derivatives | | 36200 |
| Amino acid and derivatives | N-Acetyl-l-leucine | Amino acid and derivatives | | 2320000 |
| Amino acid and derivatives | "2,6-Diaminooimelic acid" | Amino acid and derivatives | | 83300 |
| Amino acid and derivatives | L-Tyramine | Amino acid and derivatives | | 106000 |
| Amino acid and derivatives | Glutathione reduced form | Amino acid and derivatives | | 9 |
| Amino acid and derivatives | L-Isoleucine | Amino acid and derivatives | | 4750000 |
| Amino acid and derivatives | 5-Hydroxy-L-tryptophan | Amino acid and derivatives | | 92900 |
| Amino acid and derivatives | S-(methyl)glutathione | Amino acid and derivatives | | 9 |
| Amino acid and derivatives | S-(5'-Adenosy)-L-homocysteine | Amino acid and derivatives | | 9 |
| Amino acid and derivatives | N'-Formylkynurenine | Amino acid and derivatives | | 334000 |
| Amino acid and derivatives | L-Pipecolic acid | Amino acid and derivatives | | 1120000 |
| Amino acid and derivatives | L-Saccharopine | Amino acid and derivatives | | 4920000 |
| Amino acid and derivatives | L-Alanine | Amino acid and derivatives | | 18600 |
| Amino acid and derivatives | 3-N-Methyl-L-histidine | Amino acid and derivatives | | 9 |
| Amino acid and derivatives | L-Tryptophan | Amino acid and derivatives | | 65800000 |
| Amino acid and derivatives | Histamine | Amino acid and derivatives | | 25500000 |
| Amino acid and derivatives | L-Theanine | Amino acid and derivatives | | 9 |
| Amino acid and derivatives | N-Acetyl-L-tyrosine | Amino acid and derivatives | | 6120000 |
| Amino acid and derivatives | L(+)-Ornithine | Amino acid and derivatives | | 690000 |
| Amino acid and derivatives | N-Acetylaspartate | Amino acid and derivatives | | 627000 |
| Amino acid and derivatives | (5-L-Glutamyl)-L-amino acid | Amino acid and derivatives | | 386000 |
| Amino acid and derivatives | Methionine sulfoxide | Amino acid and derivatives | | 4630000 |
| Amino acid and derivatives | Dl-Norvaline | Amino acid and derivatives | | 408000000 |
| Amino acid and derivatives | Phe-Phe | Amino acid and derivatives | | 9 |
| Amino acid and derivatives | S-(5'-Adenosyl)-L-methionine | Amino acid and derivatives | | 167000 |
| Amino acid and derivatives | 4-Hydroxy-L-glutamic acid | Amino acid and derivatives | | 66900 |
| Amino acid and derivatives | L-Cystathionine | Amino acid and derivatives | | 9 |
| Amino acid and derivatives | Hexanoyl glycine | Amino acid and derivatives | | 34700 |
| Amino acid and derivatives | 3-Methylcrotonyl glycine | Amino acid and derivatives | | 9 |
| Amino acid and derivatives | L-Homocystine | Amino acid and derivatives | | 118000 |
| Amino acid and derivatives | "3-Hydroxy-3-methylpentane-1,5-dioic acid" | Amino acid and derivatives | | 8040000 |
| Amino acid and derivatives | 2-Aminoisobutyric acid | Amino acid and derivatives | | 42600000 |
| Amino acid and derivatives | "N,N-Dimethylglycine" | Amino acid and derivatives | | 10100000 |
| Amino acid and derivatives | 5-oxoproline | Amino acid and derivatives | | 195000000 |
| Amino acid and derivatives | CYS-GLY | Amino acid and derivatives | | 52300 |
| Amino acid and derivatives | N-acetylglycine | Amino acid and derivatives | | 17100000 |
| Amino acid and derivatives | N-Acetylthreonine | Amino acid and derivatives | | 460000 |
| Amino acid and derivatives | H-HomoArg-OH | Amino acid and derivatives | | 1110000 |
| Amino acid and derivatives | 1-Aminocyclopropanecarboxylic acid | Amino acid and derivatives | | 11800000 |
| Amino acid and derivatives | D-erythro-sphinganine | Amino acid and derivatives | | 68400 |
| Amino acid and derivatives | N-Acetyl-L-phenylalanine | Amino acid and derivatives | | 5990000 |
| Amino acid and derivatives | S S-Allyl-L-cysteine | Amino acid and derivatives | | 9 |
| Amino acid and derivatives | cis-4-Hydroxy-D-proline | Amino acid and derivatives | | 1120000 |
| Amino acid and derivatives | Proline | Amino acid and derivatives | | 401000000 |
| Amino acid and derivatives | Aspartic acid | Amino acid and derivatives | | 8810000 |
| Amino acid and derivatives | Glutamic acid | Amino acid and derivatives | | 16900000 |
| Amino acid and derivatives | D-(-)-Valine | Amino acid and derivatives | | 214000000 |
| Amino acid and derivatives | D-(+)-Phenylalanine | Amino acid and derivatives | | 367000000 |

**[Supplementary](https://www.mdpi.com/1422-0067/21/21/7974/htm#app1-ijms-21-07974) Table 3: Summary of RNA-seq sample data**

Sample Total Raw Reads (M) Total Clean Reads (M) Clean Reads Ratio (%)

CK-1 45.57 44.68 98.03

CK-2 45.57 44.38 97.38

CK-3 45.57 44.72 98.12

GUA-2h-1 45.57 44.83 98.36

GUA-2h-2 45.57 44.72 98.14

GUA-2h-3 45.57 44.60 97.85

GUA-24h-1 45.57 44.50 97.64

GUA-24h-2 45.57 44.48 97.60

GUA-24h-3 45.57 44.70 98.09

**[Supplementary](https://www.mdpi.com/1422-0067/21/21/7974/htm#app1-ijms-21-07974) Table 4: RNA-seq reference gene mapping data**

Sample Total Clean Reads (M) Total Mapping (%) Uniquely Mapping (%)

CK-1 44.68 83.31 78.15

CK-2 44.38 83.59 78.38

CK-3 44.72 84.04 78.89

GUA-2h-1 44.83 82.85 77.35

GUA-2h-2 44.72 83.26 77.66

GUA-2h-3 44.60 82.91 77.32

GUA-24h-1 44.50 82.94 77.51

GUA-24h-2 44.48 82.62 77.36

GUA-24h-3 44.70 84.71 79.25
