# Supplementary material for: A Novel Guanine Elicitor Stimulates Immunity in Arabidopsis and Rice by Ethylene and Jasmonic Acid Signaling Pathways
Source: Front Plant Sci. 2022 Feb 17;13:841228. doi: 10.3389/fpls.2022.841228 (PMC8893958; doi:10.3389/fpls.2022.841228)
Supplement: Supplementary file 5 [file Table_2.DOCX]

**[Supplementary](https://www.mdpi.com/1422-0067/21/21/7974/htm#app1-ijms-21-07974) Table 1: List of qRT-PCR primers used in this study**

Primers Sequence shown in 5’→3’ orientation

qPCR-OsAOX1c-F GCGTCGGATGTTCATTTTCA

qPCR-OsAOX1c-R CCGCCGTAAGATTTCTTCAGT

qPCR-OsROBHE-F GCAGAACAGAACGATCCACCA

qPCR-OsROBHE-R CCCAGTAGAAGTGTGCCCTG

qPCR-OsACO7-F ACTCGCTCATCGGAAGCAAA

qPCR-OsACO7-R GCCATGGTTTTCCACCCAGA

qPCR-OsAOS3-F CGGGCGTCGTCGGGATAG

qPCR-OsAOS3-R AACATCCACCAACCTAGTACAGTAC

qPCR-OsPR1a-F CGTCTTCATCACCTGCAACTACTC

qPCR-OsPR1a-R CATGCATAAACACGTAGCATAGCA

qPCR-OsSAMS1-F ATCATACGCCATCGGTGTCC

qPCR-OsSAMS1-R TTCCGAAGTGACCGTAAGCC

qPCR-OsWRKY53-F GTTCATCACGTCGTTCACCG

qPCR-OsWRKY53-R GTCGGGGACGCCAAGATATG

qPCR-OsOPR5-F ACAACTCGGTTGCTGATGCT

qPCR-OsOPR5-R TGTGGTGTGAATACATCTGCGT

qPCR-OsERF62-F GAGCTTCGACGACTTCCCAA

qPCR-OsERF62-R GACCAGCAGTACGAAGACCC

qPCR-OsJAZ10-F AAGCCGCGTTTTGTTTCTCG

qPCR-OsJAZ10-R TCCTCGAAGACGACCATCCT

qPCR-OsActin-F TGTATGCCAGTGGTCGTACCA

qPCR-OsActin-R CCAGCAAGGTCGAGACGAA

**[Supplementary](https://www.mdpi.com/1422-0067/21/21/7974/htm#app1-ijms-21-07974) Table 3: Summary of RNA-seq sample data**

Sample Total Raw Reads (M) Total Clean Reads (M) Clean Reads Ratio (%)

CK-1 45.57 44.68 98.03

CK-2 45.57 44.38 97.38

CK-3 45.57 44.72 98.12

GUA-2h-1 45.57 44.83 98.36

GUA-2h-2 45.57 44.72 98.14

GUA-2h-3 45.57 44.60 97.85

GUA-24h-1 45.57 44.50 97.64

GUA-24h-2 45.57 44.48 97.60

GUA-24h-3 45.57 44.70 98.09

**[Supplementary](https://www.mdpi.com/1422-0067/21/21/7974/htm#app1-ijms-21-07974) Table 4: RNA-seq reference gene mapping data**

Sample Total Clean Reads (M) Total Mapping (%) Uniquely Mapping (%)

CK-1 44.68 83.31 78.15

CK-2 44.38 83.59 78.38

CK-3 44.72 84.04 78.89

GUA-2h-1 44.83 82.85 77.35

GUA-2h-2 44.72 83.26 77.66

GUA-2h-3 44.60 82.91 77.32

GUA-24h-1 44.50 82.94 77.51

GUA-24h-2 44.48 82.62 77.36

GUA-24h-3 44.70 84.71 79.25
